# Comparative evaluation of expiratory airflow limitation between patients with COPD and BE using IOS

**DOI:** 10.1038/s41598-021-84028-9

**Published:** 2021-02-25

**Authors:** Daniele Oliveira dos Santos, Larissa Perossi, Jéssica Perossi, Letícia Helena de Souza Simoni, Mayara Holtz, Ricardo Grassi Moroli, José Antônio Baddini-Martinez, Ada Clarice Gastaldi

**Affiliations:** 1 Department of Health Sciences, Graduate Program in Functional Performance, Ribeirão Preto Medical School, University of São Paulo, Av. Bandeirantes, 3900, Monte Alegre, Ribeirão Preto, SP 14049-900 Brazil; 2 Department of Clinical Medicine, Ribeirão Preto Medical School, Ribeirão Preto, SP Brazil

**Keywords:** Outcomes research, Respiration

## Abstract

Impulse oscillometry (IOS) allows evaluation of the compartmentalized resistance and reactance of the respiratory system, distinguishing central and peripheral obstruction. The IOS measurements are getting attention in the diagnosis and differentiation of chronic respiratory diseases. However, no data are available in the literature to differentiate between COPD and BE using IOS parameters. We aimed to evaluate the feasibility of IOS in the diagnosis of bronchiectasis non-cystic fibrosis (BE) in comparison to COPD. Whole breath, inspiration, expiration, and inspiratory-expiratory difference (Δ) were evaluated based on the IOS parameters: total resistance (R5), central airway resistance (R20), peripheral airway resistance (R5-R20), reactance (X5), reactance area (AX), and resonance frequency (Fres). Fifty-nine subjects (21 Healthy, 19 BE, and 19 COPD) participated in this study. It was observed a significant difference in the comparison of healthy and pulmonary disease groups (BE and COPD) for total breathing (R5-R20, X5, AX, and Fres), inspiratory phase (R5 and R5-R5), and expiratory phase (R5-R20 and X5). The comparison between BE and COPD groups showed significant difference in the expiratory phase for resistance at 5 and 20 Hz and, ΔR5 and ΔR20**.** The IOS evidenced an increase of R5, R20 and R5-R20 in patients with BE and COPD when compared to healthy subjects. Expiratory measures of IOS revealed increased airway resistance in COPD compared to BE patients who had similar FEV1 measured by spirometry, however, further studies are needed to confirm these differences.

Chronic lung diseases lead to progressive deterioration of pulmonary function. Spirometry is the gold standard pulmonary function test to evaluate airway obstruction in pulmonary diseases^1,2^. However, spirometry parameters do not provide detailed airway assessment, but show only the severity of airflow limitation, especially large airways disorders, providing little data on the pathophysiology of the underlying disease^3^.

Other examination techniques have been used to evaluate the compartmentalized airways, such as body plethysmography and forced oscillation technique (FOT). However, plethysmography demands more expensive equipment and larger spaces, which may limit its use in clinical practice^4^. The FOT is an easy-to-apply technique that provides reliable data on respiratory mechanics by measuring pressure and flow response to small forced oscillations^5^.

The impulse oscillometry (IOS), a system developed from the FOT, measures respiratory impedance at multiple frequency ranges and allows functional evaluation of the airways by measuring the instantaneous response to pressure and flow to obtain the respiratory system impedance (Zrs), which included the respiratory resistance (*R*rs) and respiratory reactance (*X*rs) measured over a range of frequencies (usually from 3 to 35 Hz)^6^.

Zrs incorporates the in-phase and out-of-phase relationships between pressure and airflow. The in-phase component, or real part of Zrs, resistance (Rrs), is related to the resistive properties of the respiratory system. The out-of-phase or imaginary part of Zrs, reactance (Xrs), is related to elastic and inertial properties of the respiratory system^5,7^.

A major value of IOS is to differentiate central and peripheral obstruction. This system analyzes separately the total resistance of the respiratory system evaluated at 5 Hz (R5), central airways at 20 Hz (R20) and the peripheral resistance by subtracting R5-R20. In addition, the comparison between inspiratory and expiratory components can be used as markers of airflow limitation^3,8,9^. Most studies on IOS report results of the variable Rrs, especially R5 and R5-R20^3,10–12^.

According to the Global Initiative for Chronic Obstructive Lung Disease (GOLD, the severity of obstruction in COPD (GOLD I-IV) is assessed by spirometry, with the forced expiratory volume in the first second (FEV_1_)^13^. In BE multifunctional indexes as Bronchiectasis Severity Index and FACED^9,14^, the FEV_1_ is also included to evaluate airflow obstruction, but the severity level of BE is defined only by FEV_1_.

This functional parameter is not enough to differentiate COPD from BE once the airflow obstruction severity did not indicate the degree of airway damage. The purpose of the present study was to evaluate the respiratory system of COPD and bronchiectasis subjects using IOS parameters to differentiate the diseases and, to compare both groups with healthy controls.

## Methods

### Design

The current work is an observational cross-sectional study performed in accordance with STROBE, and the data was prospectively collected.

### Patients

A total of 59 patients were evaluated: 21 with no pulmonary disease (Healthy), 19 with bronchiectasis (BE), and 19 with chronic obstructive pulmonary disease (COPD). The data from patients with BE and Healthy were collected at the Laboratory for Assessment of Respiratory System (LAR) of Ribeirão Preto Medical School, and volunteers with COPD were evaluated at the Imperial College London. The data were collected from January 2013 to September 2016 following the same protocol. Patients with upper respiratory tract disease treated with antibiotics in the four weeks prior to the study, presence of hemoptysis or history of pulmonary surgery were excluded. All subjects signed the form of Informed Consent. The Ethics Committee of both institutions, the HC-FMRP Research Ethics Committee (6007/2007) and the National Research Ethics Service, United Kingdom (13/LO/0339) approved the current study. The healthy subjects included had no complaints or history of lung disease and demonstrated normal values of spirometry.

### Spirometry

All patients underwent spirometry to detect respiratory changes using the Jaeger MasterScreen spirometer (Jaeger Co, Wurzburg, Germany), according to the ATS/ERS guidelines^2^.

### Impulse oscillometry

The impedance of the respiratory system was evaluated using the Jaeger MasterScreen IOS (Jaeger Co, Wurzburg, Germany), according to the methodology described by Oostveen and colleagues^5^. The following parameters were evaluated: resistance (KPa/L/s) at frequencies of 5 Hz and 20 Hz (R5, R20, and R5-R20); reactance (KPa/L/s) at a frequency of 5 Hz(X5); resonance frequency (Fres) in Hz; and reactance area (AX) in KPa/L during total breathing, inspiratory, and expiratory phases. The difference in inspiratory-expiratory value was also calculated to obtain the resistance (ΔR5, ΔR20, and ΔR5-R20) and reactance (ΔX5). The predicted values were calculated by age-corrected^15^.

### Statistics

A sample calculation was made for the R5 variable, based on a previous study^16^, with a difference of 0.04, standard deviation of 0.04 and power of 80%, resulting in 19 volunteers per group. The software R Core Team (version 3.4.3, Vienna, Austria, 2017) and the SAS Statistical Software (version 9.3, SAS Institute, Inc. Cary, NC) were used to analyze the data. The one-way ANOVA with Levene’s pre-test of homogeneity of variance was used for comparison between the groups. Duncan's post-test of multiple comparisons was used for comparison of the differences. Statistical significance was considered when p value was < 0.05.

### Ethics approval and consent to participate

This work was approved by HC-FMRP Research Ethics Committee (6007/2007) and the National Research Ethics Service, United Kingdom (13/LO/0339).

## Results

The data of sample characterization are shown in Table 1. The comparison between groups is shown in Table 2, there were more significant comparisons in the comparisons with healthy than between BE and COPD.

**Table 1:** Patient characterization.

	Healthy (n = 20)	BE (n = 19)	COPD (n = 19)
**Anthropometric data**			
Age (years)	57 ± 12	57 ± 14	67 ± 7
Sex (M/F)	4/16	9/10	5/14
BMI (kg/m^2^)	24.2 ± 8	24.1 ± 4.6	24.9 ± 4.4
**Pulmonary function**			
FEV_1_ (%)	103 ± 14	60 ± 28	61 ± 18
FVC (%)	107 ± 15	79 ± 24	105 ± 18
FEV_1_/FVC (%)	97 ± 6	63 ± 17	60 ± 14

M: male; F: female; BMI: body mass index.

FEV_**1**_ (%): forced expiratory volume in the first second.

FVC: forced vital capacity; FEV_**1**_/FVC (%): Tiffeneau index.

Healthy: no pulmonary disease; BE: bronchiectasis.

COPD: chronic obstructive pulmonary disease.

**Table 2:** Values of IOS parameters in all breathing phases.

IOS parameters	Healthy	BE	COPD	*p* value
**R5 (kPa/l/s)**				
Predict value*	0.35 ± 0,09	0.33 ± 0.08	0.32 ± 0.07	–
Total breathing	0.41 ± 0.12	0.50 ± 0.20	0.60 ± 0.13	< 0.001^b^
Inspiratory phase	0.35 ± 0.09	0.45 ± 0.17	0.49 ± 0.11	< 0.05^a^; ^<^0.01^b^
Expiratory phase	0.47 ± 0.16	0.53 ± 0.21	0.69 ± 0.17	< 0.001^b^; 0.01^c^
Δ R5	− 0.11 ± 1.08	− 0.07 ± 2.25	− 0.20 ± 1.20	< 0.01^b^; < 0.001^c^
**R20 (kPa/l/s)**				
Predict value*	0.33 ± 0.04	0.31 ± 0.05	0.31 ± 0.06	–
Total breathing	0.34 ± 0.08	0.34 ± 0.11	0.41 ± 0.10	ns
Inspiratory phase	0.30 ± 0.07	0.31 ± 0.09	0.37 ± 0.09	< 0.05^b^
Expiratory phase	0.37 ± 0.10	0.35 ± 0.13	0.44 ± 0.12	0.05^c^
Δ R20	− 0.07 ± 4.82	− 0.03 ± 9.01	− 0.07 ± 5.99	ns
**R5 – R20 (kPa/l/s)**				
Total breathing	0.07 ± 0.06	0.16 ± 0.13	0.20 ± 0.08	0.01^a^; < 0.001^b^
Inspiratory phase	0.05 ± 0.04	0.14 ± 0.13	0.12 ± 0.06	< 0.01^a^; < 0.05^b^
Expiratory phase	0.09 ± 0.09	0.18 ± 0.14	0.25 ± 0.10	0.05^a^; < 0.001^b^
Δ R5–R20	− 0.04 ± 0.08	− 0.03 ± 0.11	− 0.13 ± 0.15	< 0.001^b^; 0.0001^c^
**X5 (kPa/l/s)**				
Predict value*	− 0.14 ± 0.02	− 0.14 ± 0.03	− 0.14 ± 0.03	–
Total breathing	− 0.11 ± 0.15	− 0.27 ± 0.16	− 0.26 ± 0.11	< 0.01^a,b^
Inspiratory phase	− 0.14 ± 0.07	− 0.23 ± 0.11	− 0.21 ± 0.07	< 0.01^a^
Expiratory phase	− 0.17 ± 0.14	− 0.30 ± 0.21	− 0.32 ± 0.15	< 0.05^a,b^
Δ X5	0.02 ± 0.08	0.06 ± 0.11	0.11 ± 0.15	ns
AX	0.90 ± 1.08	2.40 ± 2.26	2.33 ± 1.20	0.01^a,b^
Fres	16.50 ± 4.82	24.50 ± 9.01	24.27 ± 6.00	0.001^a,b^

^a^Difference between Healthy and BE groups; ^b^ difference between Healthy and COPD groups;

^c^Difference between BE and COPD groups.

R5: total resistance; R20: central resistance; R5–R20: peripheral resistance; X5: reactance; AX: reactance area; Fres: resonance frequency.

Healthy: no pulmonary disease; BE: bronchiectasis; COPD: chronic obstructive pulmonary disease.

*p* value: Duncan post-hoc test.

Predicted values*: calculated by age corrected, Oostveen et al.^15^.

### Total breathing analysis (Rrs and Xrs)

Comparison between Healthy and chronic pulmonary disease groups showed increased of R5 in the COPD group (*p* < 0.001), R5-20 in the BE (*p* = 0.01) and COPD (*p* = 0.001) groups; greater X5 negativity in the BE (*p* = 0.01) and COPD (*p* = 0.01) groups; increased AX in the BE (*p* = 0.01) and COPD (*p* = 0.01) groups, and increased Fres in the BE (*p* = 0.001) and COPD (*p* = 0.001) (Table 2).

**Figure 1:**
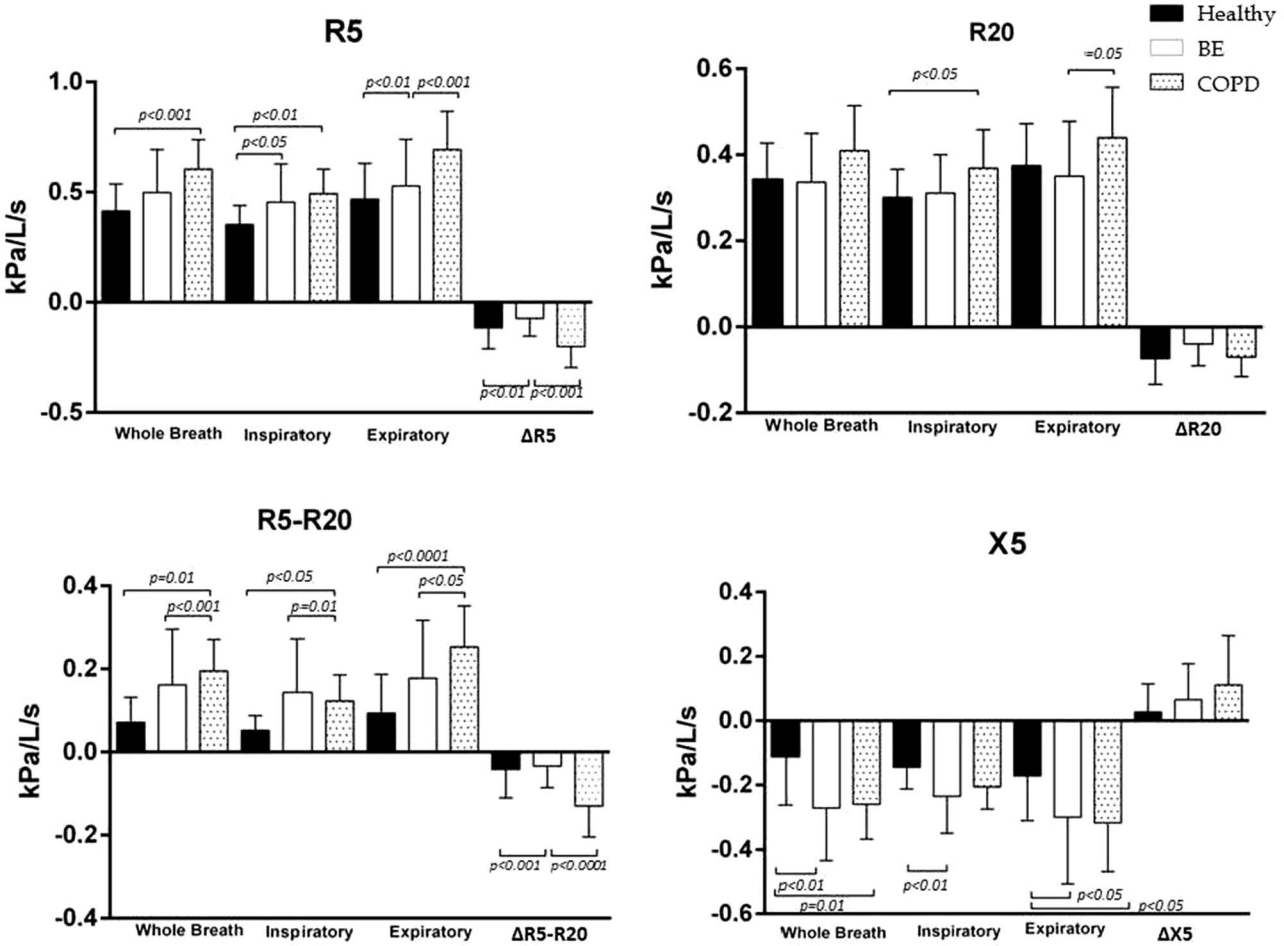
Comparison of resistance at 5 Hz (R5), R20, R5-R20 and reactance at 5 Hz (X5) in whole-breath, inspiratory and expiratory phases and difference inspiratory–expiratory (Δ), in healthy subjects (back bars), patients with bronchiectasis (BE) (white bars) and chronic obstructive pulmonary disease (COPD) (dotted bar).

**Figure 2:**
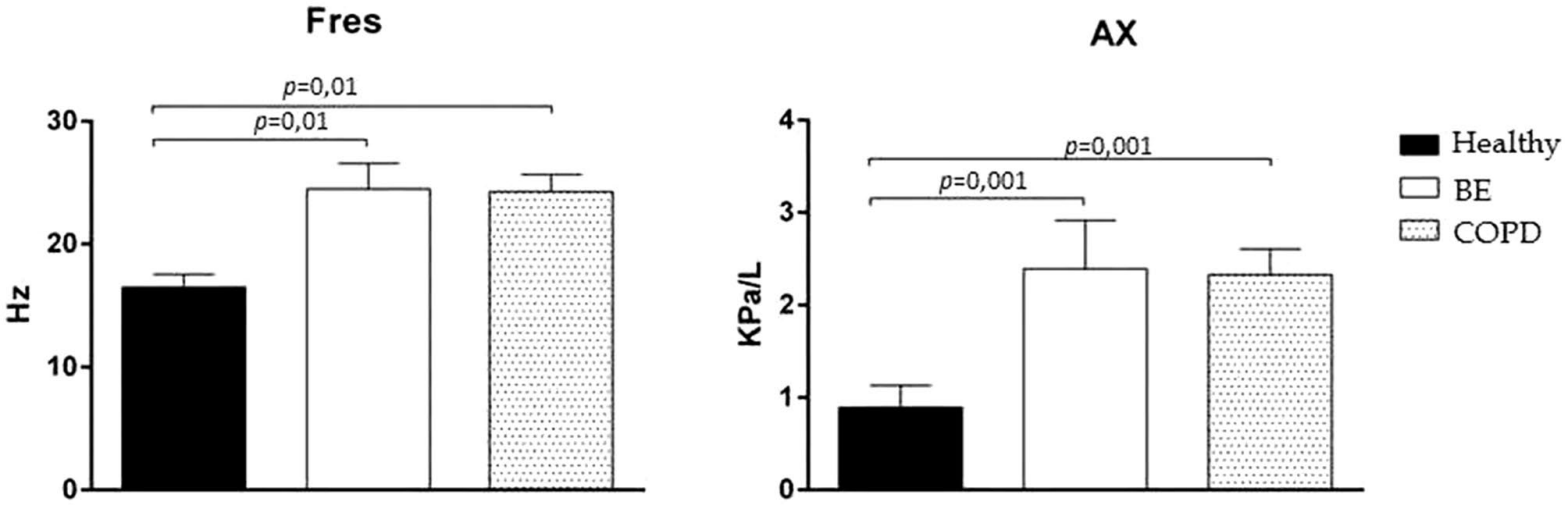
Comparison of resonant frequency (Fres) and rectance area (Ax) in whole-breath, in healthy subjects (black bars), patients with bronchiectasis (BE) (white bars) and chronic obstructive pulmonary disease (COPD) (dotted bar).

When compared the BE and COPD groups showed no statistically significant difference in any of the assessed variables (Figs. 1, 2).

### Inspiratory phase analysis

In comparison between Healthy and chronic pulmonary disease groups were observed an increase of R5 in the BE (*p* < 0.05) and COPD (*p* < 0.01) groups, R20 in the COPD group (*p* < 0.05), R5-R20 in the BE (*p* < 0.01) and COPD (*p* < 0.05) groups; and greater X5 negativity in the COPD group (*p* < 0.01) (Table 2).

Comparison between the BE and COPD groups showed no statistically significant difference in any of the assessed variables (Fig. 1).

### Expiratory phase analysis

When healthy and chronic pulmonary disease groups were compared, we observed an increase of R5 in the COPD group (*p* < 0.001); R5–R20 in the BE (*p* = 0.05) and COPD (*p* < 0.001) groups; and greater X5 negativity the BE (*p* < 0.05) and COPD (*p* < 0.05) (Fig. 1, Table 2).

Comparison between the pulmonary disease groups showed increased R5 (*p* = 0.01) and R20 (*p* = 0.05) in the COPD group (Fig. 1).

### Inspiratory-expiratory difference

The comparison between the Healthy group and chronic pulmonary diseases group showed an increase in COPD group in ΔR5 (*p* < 0.01) and ΔR5-R20 (*p* < 0.001). No significant difference in ΔR20 and ΔX5 was observed in any comparison (Fig. 1, Table 2).

Comparison between the pulmonary disease groups showed increased values of ΔR5 (*p* < 0.001) and ΔR5-R20 (*p* = 0.0001). ΔR20 and ΔX5 analyses showed no significant difference between the groups (Fig. 1).

## Discussion

The current study evaluated respiratory mechanics of volunteers healthy, with BE and COPD by IOS. It was found that patients with chronic respiratory diseases have higher resistance values of the respiratory system when compared to healthy subjects, as previously described in the literature. However, impairment in expiratory resistance was more pronounced in patients with COPD, which indicates greater dynamic limitation of expiratory airflow when compared to patients with BE. This differentiation demonstrated by IOS is the major finding of the current study, which was not shown by spirometry and has not been demonstrated in previous studies.

In COPD, major limitation of expiratory flow occurs due to closing of small airways, mainly due to remodeling of these structures. This change affects Rrs, causing difficulty to breath in and out. Another important factor is the loss of lung elasticity due to destruction of the respiratory units, alveolar duct, and alveoli, which impair the release of air from the lungs, thereby increasing airway resistance. Clinically, changes in respiratory pattern, dynamic hyperinflation, and dyspnea are evident^17^. Crisafulli and collaborators reported the progressive increase in small airway resistance related to the severity of the disease in individuals with COPD assessed by the IOS, these changes may be closely related by the symptoms^18^.

In BE, small airway disorders often precede large airway disorders. The severity of the condition is associated with the magnitude of airway inflammation and remodeling which leads to increased resistance^19–22^.

In this study, the compartmentalized impedance analysis and the different phases of respiration showed that in both diseases there is impairment of the small airways, evidenced by the increase in peripheral resistance in total respiration and in the inspiratory and expiratory phases. However, the ΔR5-R20 analysis showed a dynamic impairment in COPD.

Although the literature suggests that there is a small contribution by oscillometry to the diagnosis once the disease is established, this technique may be useful to identify early changes. Su et al., reported that associating IOS with spirometry contributes to an additional increase in the diagnostic value to identify small-airway disorders in early-stage COPD, once IOS was more sensitive in detecting small airways’ obstruction^23^. In addition, the IOS was able to detect minor changes in treatment in patients with COPD^16,24^ and bronchiectasis^11^.

The results of the present study indicate that oscillometry, which allows a compartmentalized analysis using data from small airways and inspiratory and expiratory phases, can contribute to diagnostic differentiation, suggesting more precise interventions and individualized treatment. It is still possible to differentiate COPD from BE by radiologic approaches^25^. However, IOS assessments do not expose the subjects to radiological effects.

Paredi et al. demonstrated that reactance (Xrs) values could be used to differentiate asthma from COPD^3^, since this variable is directly correlated with changes in the limitation of expiratory flow. Johnson et al. concluded that Xrs predicts transpulmonary resistance more accurately than Rrs^22^. The current study also showed that the X5 analysis distinguished healthy subjects from patients with BE and COPD in the total breath and in expiratory phase, and in the inspiratory phase only the BE group differed from the healthy group. However, there was no difference between bronchiectasis and COPD in X5 analysis. One possible explanation is that the limitation of flow in BE and COPD is due to peripheral airway involvement.

Additionally, another variable related to flow limitation, ΔR5-R20, showed greater increase in patients with COPD when compared to BE. This occurrence may also be confirmed by the higher values ​​of total resistance in the expiratory phase obtained in the COPD group, showing a dynamic limitation of the expiratory flow in these patients, and suggesting that the alterations observed in volunteers with BE may mainly be due to structural alterations in bronchi and bronchioles and accumulation of secretions.

In relation to the use of other IOS parameters to differentiate respiratory system diseases, Guan et al. found a difference between BE and healthy subjects by resonant frequency values (Fres)^10^. In the current study, BE and COPD subjects presented greater values of Fres when compared with healthy volunteers. Studies have reported that Fres reflects the inertial properties of the airways and the pulmonary peripheral capacitance, while R5-R20 indicates the caliber of small airways and ventilation heterogeneity. However, no difference in Fres was observed between the respiratory diseases investigated in the current study.

Although both diseases mainly affected the peripheral airways, in the current study, patients with COPD presented higher values ​​of total resistance in total breathing and in the expiratory phase, and of central resistance during inspiration. Both respiratory diseases showed increased peripheral resistance in total breathing and in the inspiratory and expiratory phases when compared to healthy subjects.

The delta analysis differentiated COPD participants from the BE and Healthy. Paredi et al., analyzed the difference between inspiration and expiration in R5 in patients with asthma and COPD and reported that in cases of low airway resistance, the airway pressure gradient between the mouth and the alveoli is small and this dynamic effect is small. In addition, the study reported that the inspiratory-expiratory difference in resistance is low in healthy subjects. However, with an increase in the airway resistance, an increase in the inspiratory-respiratory differences in isovolume transmural pressures is observed and the dynamic effects are more obvious^3^.

As a limitation, the mean age of bronchiectasis and COPD groups were 57 ± 14 and 67 ± 7 years, respectively, however, in the literature, there is no prediction equation specific for this population. To avoid a possible confounder and to confirm the differences between these groups, we calculated the predicted values by age-correction described in the literature^15^.

## Conclusion

The assessment by IOS evidenced an increase of respiratory system resistance in patients with chronic respiratory diseases when compared to healthy subjects. Comparing the whole breath, inspiratory and expiratory measures between diseases, there is a greater impairment of expiratory resistance in COPD, suggesting that IOS might better reveal expiratory airflow limitation than spirometry in patients who had similar FEV_1_ values, however, further studies are needed to confirm these differences.

## Availability of data and material

The datasets generated and/or analyzed during the current study are not publicly available due to the availability of the corresponding author.

## Abbreviations

IOS: Impulse oscillometry system
COPD: Chronic obstructive pulmonary disease
R5: Total airway resistance
R20: Central airway resistance
R5-R20: Peripheral airway resistance
X5: Respiratory reactance
AX: Reactance area
FRES: Resonant frequency
BE: Bronchiectasis
FOT: Forced oscillation technique
Zrs: Respiratory impedance
Xrs: Respiratory resistance
GOLD: Global Initiative for Chronic Obstructive Lung Disease
FEV_1_: Forced expiratory volume in first second
LAR: Laboratory of Assessment Respiratory
HC-FMRP: Hospital of Clinics of Ribeirao Preto Medical School
Hz: Hertz
